# Thyroid tuberculosis: Rare case report

**DOI:** 10.1016/j.radcr.2025.08.024

**Published:** 2025-09-16

**Authors:** Chaimae Abourak, Siham Oukassem, Wadie Zouita, kenza Bentalha, Meriem Boubekri, Zakia Bernoussi, Kaoutar Znati, taha yassine Aaboudech, Wafae Elaamadi, soumaya Elyoussoufi, Abdellah Achir, Bilal Motassim billah, Ittimade Nassar, Kaoutar Imrani

**Affiliations:** a Central Radiology Department, Ibn Sina University Hospital, Faculty of Medicine and Pharmacy of Rabat, Mohammed V University, Rabat, 10000, Morocco; b Anatomopathology Department, Ibn Sina University Hospital, Faculty of Medicine and Pharmacy of Rabat, Mohammed V University, Rabat, 10000, Morocco; c Thoracic Surgery Department, Ibn Sina University Hospital, Faculty of Medicine and Pharmacy of Rabat, Mohammed V University, Rabat, 10000, Morocco

**Keywords:** Thyroid gland, Tuberculosis, Thyroid tuberculosis, Thyroid abscess, Medical imaging, Ultrasonography, Computed tomography

## Abstract

Tuberculosis remains a major public health issue, particularly in developing countries where it is endemic, such as Morocco. While pulmonary tuberculosis is the most common form, extrapulmonary involvement can affect various organs, including the thyroid gland, although this localization is exceptionally rare. We report the case of a middle-aged Moroccan man diagnosed with a tuberculous thyroid abscess, an uncommon and misleading presentation. The diagnosis was confirmed by cytobacteriological and histopathological analysis of a thyroid specimen. This case underscores the need to consider tuberculosis in the differential diagnosis of atypical cervical masses, especially in endemic regions.

## Introduction

Thyroid tuberculosis (TTB) is an exceptionally rare condition, even in regions with a high prevalence of tuberculosis [[1], [2], [3], [4]]. According to the World Health Organization, tuberculosis remains a major global public health concern, with millions of new cases each year, of which approximately 15 to 20% involve extrapulmonary forms that often pose significant diagnostic challenges. The first report of thyroid tuberculosis dates back to 1862, when Lebert described the case of a young woman who died from miliary tuberculosis [5]. Diagnosing TTB remains difficult due to the lack of specific clinical signs. Its clinical spectrum is broad, ranging from asymptomatic involvement to obvious clinical manifestations, presenting as an isolated thyroid nodule, diffuse or multinodular goiter [3]. Thyroid abscess is the rarest clinical presentation [4]. We report here an atypical case of a tuberculous thyroid abscess diagnosed in a middle-aged Moroccan man.

## Case report

A 46-year-old Moroccan man with no significant medical history, including no personal or family history of tuberculosis and no recent travel to hyperendemic areas, presented to the emergency department with a rapidly progressing swelling on the left side of his neck over 2 weeks. The swelling was associated with severe local pain, fever (38.2°C), moderate dyspnea, and progressive dysphagia. The patient denied any chronic respiratory symptoms or significant weight loss.

On physical examination, the patient was febrile and exhibited mild dyspnea at rest. Palpation revealed a firm, tender, and large mass measuring approximately 6 cm in diameter located at the left thyroid lobe. The overlying skin was indurated and slightly erythematous with a tense appearance, but no frank signs of inflammation or fluctuance were observed. The mass exhibited limited mobility. No palpable cervical lymphadenopathy was noted. Pulmonary auscultation revealed crackles in the left upper lung field. The rest of the clinical examination was unremarkable.

Laboratory tests showed a normal complete blood count (CBC) with mild lymphocytosis at 4,200/mm³ (normal range: 1000-4000), an elevated erythrocyte sedimentation rate (ESR) of 48 mm in the first hour, and a C-reactive protein (CRP) level of 28 mg/L. Thyroid function tests were within normal limits: thyroid-stimulating hormone (TSH) at 2.1 µIU/mL (reference range: 0.4-4.0) and free thyroxine (T4) at 12 pmol/L (reference range: 9-20). HIV serology was negative. Chest X-ray revealed mild bronchial thickening without obvious infiltration.

**Fig. 1:**
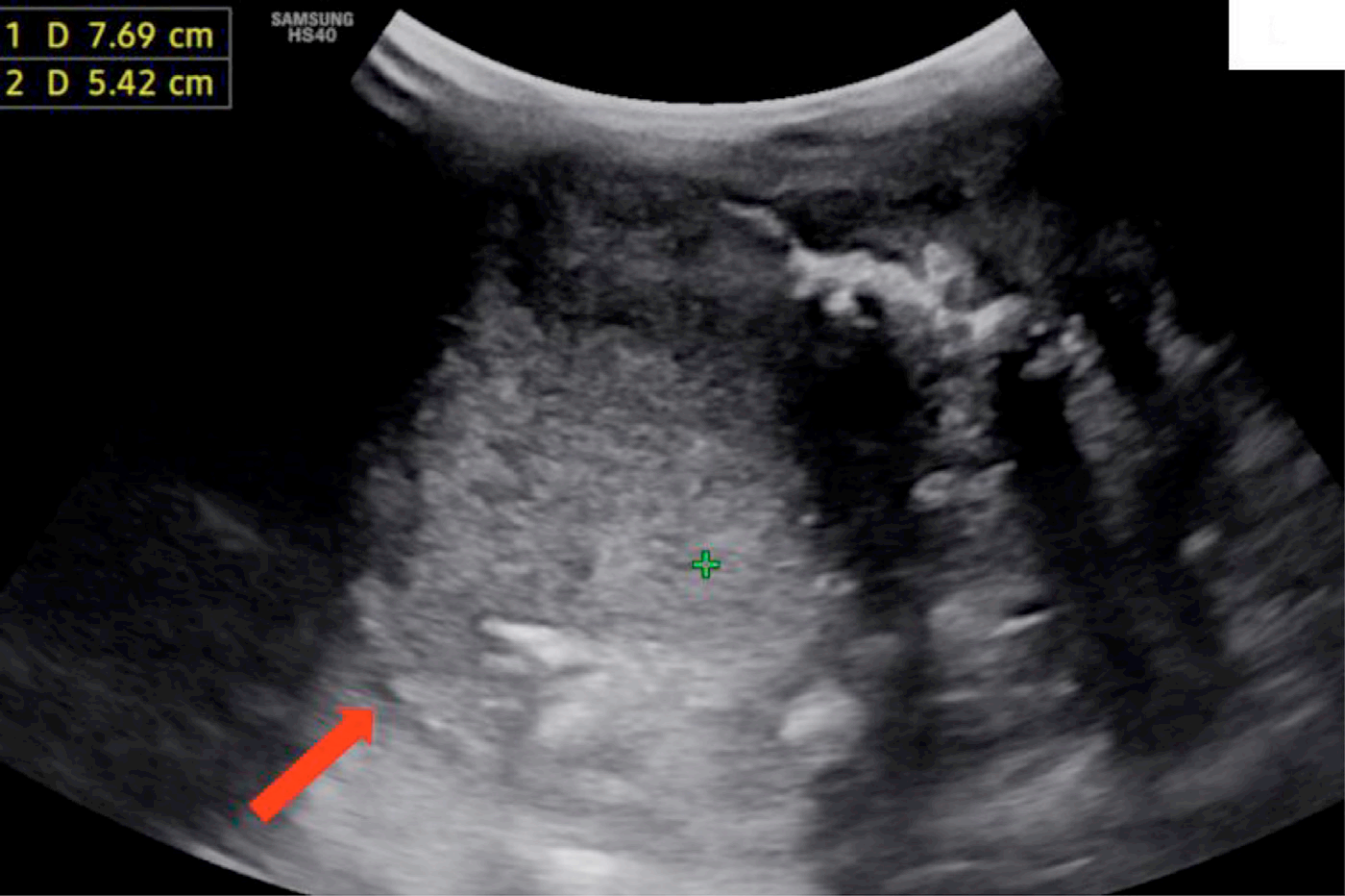
Cervical ultrasound, axial view: A collection involving the left thyroid lobe with echogenic content containing calcifications, generating posterior acoustic shadowing (red arrow).

Cervical ultrasound showed a large, hyperechoic cystic collection measuring approximately 5.5 × 4 cm at the lower pole of the left thyroid lobe, with a regular wall, internal mobile echoes, and calcifications (Fig. 1). The isthmus and right thyroid lobe were normal without cervical lymphadenopathy.

**Fig. 2:**
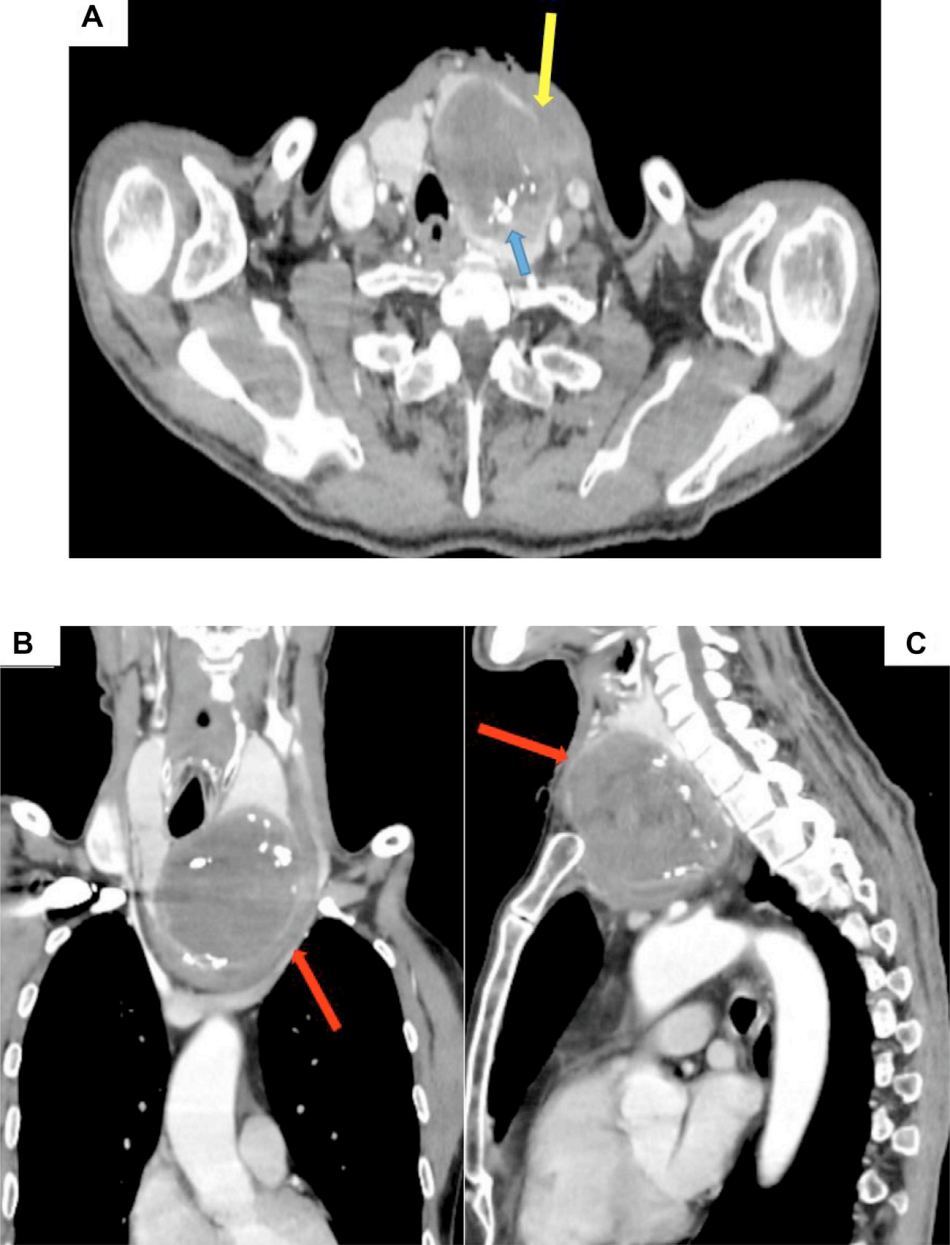
Cervicothoracic CT scan. Axial (A), sagittal (B), and coronal (C) reconstructions. showing a plunging left goiter with a large inferior polar collection (81 × 89 × 83 mm) (red arrow),containing calcifications (blue arrow). and no postcontrast enhancement, associated with a fistulous tract extending to the subcutaneous plane with soft tissue infiltration (yellow arrow), displacing the tracheoesophageal axis posteriorly, the left sternocleidomastoid muscle laterally, and extending into the anterior superior mediastinum, while exerting a mass effect on the left internal jugular vein and brachiocephalic veins, which remain patent, without direct contact with the brachiocephalic trunk or right subclavian artery.

**Fig. 3:**
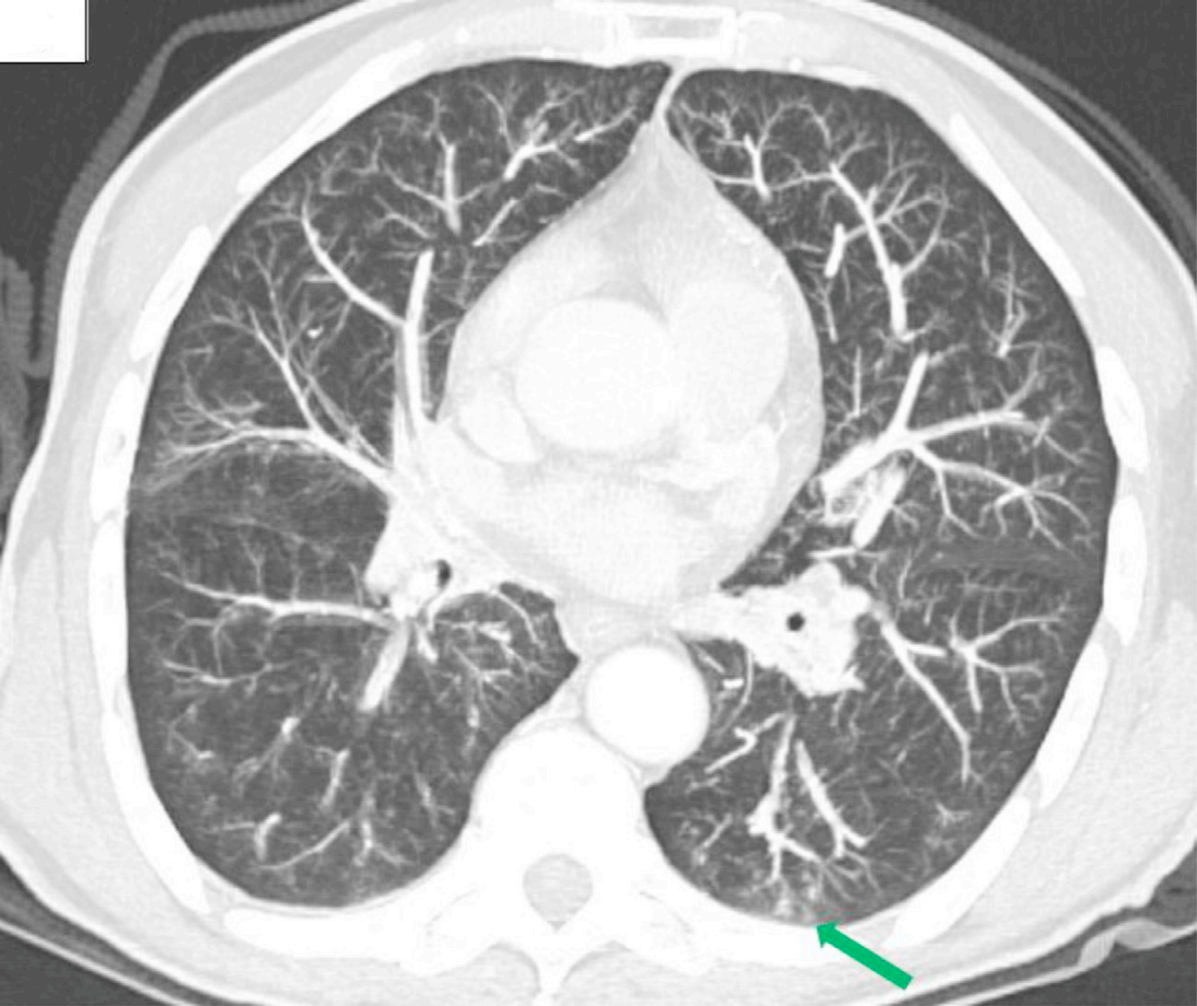
Thoracic CT scan. Axial slice in lung window. A cluster of bronchiolar micronodules forming a tree-in-bud pattern in the left Fowler, suggestive of an infectious bronchiolitis focus (green arrow).

Cervicothoracic computed tomography (CT) confirmed a large hypodense collection with a thickened, enhancing wall containing internal calcifications. This collection extended from the lower pole of the left thyroid lobe to the anterior mediastinum via the cervicothoracic inlet. A fistulous tract communicating with the overlying subcutaneous tissue was identified (Figs. 2A, B and C and Fig. 3). Additionally, multiple micronodules displaying a “tree-in-bud” pattern were observed in the left upper lung lobe, consistent with active pulmonary tuberculosis.

Based on these clinical and radiological findings, ultrasound-guided fine-needle aspiration was performed, yielding purulent material in which acid-fast bacilli (AFB) were detected on direct examination. Culture confirmed the presence of Mycobacterium tuberculosis.

**Fig. 4:**
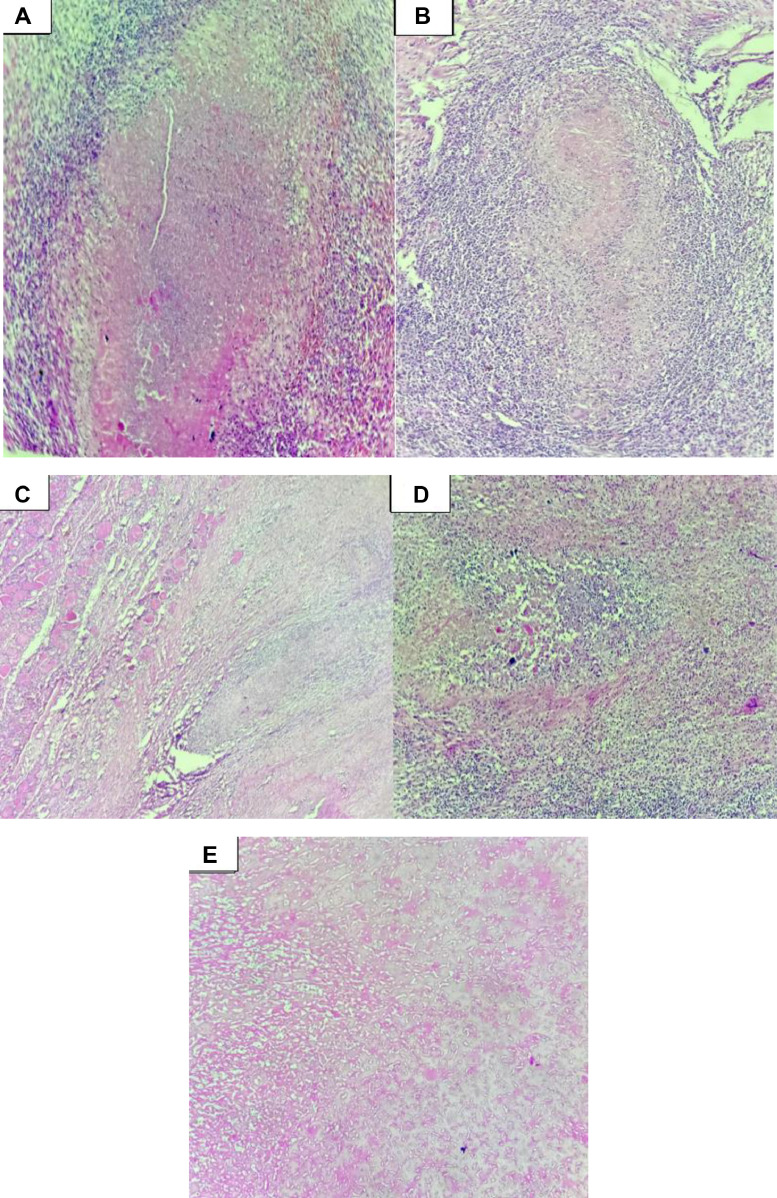
(A-E) Histopathological examination of the thyroid tissue. The thyroid parenchyma is extensively replaced by necrotic areas associated with granulation tissue composed of clusters of histiocytes mixed with polymorphous inflammatory cells. Multiple, discrete, variably sized round granulomas are present, characterized by a rim of epithelioid cells surrounding extensive eosinophilic necrosis, sometimes suppurative, containing cellular debris. Additional features include hyalinized fibrous remodeling, calcifications, and cholesterol crystals. The fistulous tract exhibits acute inflammatory changes with granulation tissue formation. Staining: Hematoxylin and Eosin (H&E). Magnifications vary across images to highlight both tissue architecture and cellular details.

Due to the volume and deep extension of the collection, a left isthmolobectomy combined with fistulectomy was performed. Macroscopic examination revealed a heterogeneous thyroid gland without distinct nodules, showing hemorrhagic, necrotic, fibrous areas, and calcifications, along with a thickened peripheral capsule. Histopathological analysis confirmed caseofollicular thyroid tuberculosis without malignancy. The fistulous tract exhibited nonspecific acute inflammation (Figs. 4 A-E).

The patient was started on standard first-line antituberculous therapy comprising isoniazid (5 mg/kg/day), rifampicin (10 mg/kg/day), pyrazinamide (25 mg/kg/day), and ethambutol (15 mg/kg/day) for 2 months (intensive phase), followed by a 4-month continuation phase with isoniazid and rifampicin. The treatment was well tolerated without major side effects. Clinical, biological (including CBC and liver function tests), and radiological monitoring were regularly performed. After 6 months, the patient showed complete clinical resolution of symptoms and significant radiological improvement of the pulmonary micronodules.

## Discussion

Tuberculosis (TB) remains a major public health concern worldwide, particularly in developing countries, despite significant advances in prevention and management. Extrapulmonary tuberculosis (EPTB) presents a diagnostic challenge due to its rarity in certain anatomical locations, such as the thyroid gland [3]. Even in endemic countries like Morocco, thyroid involvement remains exceptional [6,7]. Typically discovered incidentally, this rare localization can be difficult to diagnose in the absence of specific clinical or biological signs [7]. This diagnostic challenge is particularly relevant in endemic areas where thyroid tuberculosis (TTB) remains an unusual entity [7].

Several hypotheses have been proposed to explain the rarity of TTB, including the bactericidal properties of thyroid colloid, high vascularity, excess iodine content, and increased phagocytic activity in hyperthyroid states [4,5]. Mycobacterium tuberculosis can reach the thyroid through hematogenous spread or by contiguous extension from adjacent lymphatic foci, such as cervical or mediastinal lymph nodes [4,5].

A review of reported cases in the literature reveals a female predominance (44 women versus 32 men), with the majority of cases occurring in middle-aged women [8]. Our case involving a 46-year-old man is therefore somewhat atypical but aligns with previously reported male cases.

TTB can present in 5 pathological forms: disseminated miliary foci, caseating goiter, fistulized cold abscess, chronic fibrosing form mimicking De Quervain’s thyroiditis, and acute abscess [4]. Our patient presented with a fistulized cold abscess, 1 of the rarest clinical manifestations [2].

The nonspecific symptoms of TTB often delay diagnosis. It is frequently discovered incidentally during pathological examination or imaging studies, and can be mistaken for other thyroid diseases. Ultrasound and ultrasound-guided fine-needle aspiration biopsy (FNAB) are critical for accurate diagnosis and to prevent unnecessary thyroidectomy [4,5]. In our patient, nonspecific general symptoms such as fever and pain, combined with compressive manifestations, prompted further imaging and diagnostic workup.

Ultrasound features of TTB remain poorly described due to the rarity of cases. Focal nodular TTB may mimic thyroid carcinoma, while acute tuberculous abscesses can resemble bacterial suppurative abscesses [4,5]. Few reports detail the radiological characteristics of thyroid tuberculous abscesses. Thyroid scintigraphy may show hypo- or hyperfixation depending on lesion type, but is generally nonspecific. Computed tomography (CT) can demonstrate nodular lesions, abscesses, or calcifications, but findings are also not pathognomonic. The presence of tuberculosis elsewhere in the body is a valuable diagnostic clue [4,5].

In our case, imaging revealed a large collection in the left thyroid lobe with a cutaneous fistulous tract. CT clarified the extent and anatomical relationships of the collection, revealed calcifications, and identified an infectious bronchiolitis focus consistent with active pulmonary TB. This pulmonary involvement, seen as micronodules with a tree-in-bud pattern on CT, further supports the diagnosis and emphasizes the need to search for concomitant TB foci [6,7].

Biological markers such as erythrocyte sedimentation rate (ESR) and C-reactive protein (CRP) help assess systemic inflammation. FNAB remains indispensable for cytological and bacteriological confirmation, revealing epithelioid granulomas with central caseous necrosis. Acid-fast bacilli detection by Ziehl-Neelsen staining or culture confirms diagnosis but may not always be necessary in endemic regions [5]. In our patient, acid-fast bacilli were identified, confirming TTB.

The differential diagnosis of TTB includes infectious thyroiditis, subacute granulomatous thyroiditis, fungal infections, sarcoidosis, granulomatous vasculitis, foreign body reactions, and thyroid malignancies, the latter sometimes coexisting with tuberculosis [5].

Thyroid malignancy may present as a nodular mass or goiter, with or without compressive symptoms, and can be associated with intralesional calcifications, complicating imaging interpretation. Histological confirmation through fine-needle aspiration biopsy (FNAB) or surgical biopsy is essential to rule out or confirm cancer.

Bacterial abscesses typically occur in the context of acute infection, often with marked inflammatory signs (redness, warmth, severe pain) and predisposing factors such as immunosuppression or underlying diseases. Bacteriological cultures help identify causative organisms distinct from Mycobacterium tuberculosis.

Sarcoidosis, a systemic granulomatous disease, can rarely involve the thyroid gland. It is characterized histologically by noncaseating granulomas, which differentiate it from tuberculosis, where caseous necrosis is a hallmark.

In our case, the combination of clinical and radiological findings, along with the identification of acid-fast bacilli on direct examination of the fine-needle aspiration specimen and histological confirmation of granulomas with caseous necrosis, allowed us to exclude other diagnoses and confirm thyroid tuberculosis.

Treatment primarily relies on antituberculous chemotherapy, typically a 6-month regimen with isoniazid, rifampicin, pyrazinamide, and ethambutol, leading to favorable outcomes in most cases [5,7]. Surgical intervention is reserved for large abscesses or when diagnosis cannot be confirmed by FNAB [4,5]. Our patient required a left isthmo-lobectomy and fistulectomy due to the collection’s size and fistula.

Therapeutic failure is rare and often linked to bacterial resistance. A thorough search for other TB sites is essential to optimize management and prevent relapses, which are more difficult to treat [[5], [6], [7]].

Several recent case reports highlight similar presentations of tuberculous thyroid abscesses, underscoring the diagnostic complexity and the importance of considering TTB in endemic regions[9,10]. For instance, Kumar et al. [9] described a comparable case of thyroid abscess in a middle-aged man successfully treated with surgery and anti-TB therapy, reinforcing the clinical and therapeutic approach we applied. Another case by Gupta et al. [10] emphasized the diagnostic role of FNAB in avoiding unnecessary extensive surgery.

## Conclusion

With the global increase in tuberculosis incidence, the unusual presentation of extrapulmonary forms presents a diagnostic challenge, requiring heightened vigilance and thorough evaluation. Early diagnosis significantly improves prognosis. Therefore, thyroid tuberculosis should be considered in the differential diagnosis of thyroid nodular lesions, particularly in patients living in areas with high tuberculosis endemicity.

The diagnosis of a thyroid tuberculous abscess is rarely based solely on clinical findings. A history of tuberculosis, a known tuberculous focus elsewhere in the body, the presence of cervical lymphadenopathy, or an elevated erythrocyte sedimentation rate may suggest the diagnosis, but thyroid tuberculosis can occur even in the absence of these factors. Ultrasound and ultrasound-guided fine-needle aspiration allow for an accurate diagnosis, thus avoiding unnecessary thyroidectomy, as these patients respond very well to antituberculosis treatment.

## Patient consent

Written informed consent for the publication of this case report was obtained from the patient
